# Cross-Lagged Relationships between Morphological Awareness and Reading Comprehension among Chinese Children

**DOI:** 10.3389/fpsyg.2016.01379

**Published:** 2016-09-21

**Authors:** Yahua Cheng, Jie Zhang, Xinchun Wu, Hongyun Liu, Hong Li

**Affiliations:** ^1^Department of Psychology, Ningbo UniversityNingbo, China; ^2^Research Center of Children’s Reading and Learning, Beijing Key Laboratory of Applied Experimental Psychology, School of Psychology, Beijing Normal UniversityBeijing, China; ^3^College of Education, University of Houston, HoustonTX, USA

**Keywords:** morphological awareness, reading comprehension, word reading, longitudinal cross-lagged study, Chinese children

## Abstract

The present study examined the developmental relationship between morphological awareness (MA) and reading comprehension (RC) using a 2-year and four-wave cross-lagged design with a sample of 149 Chinese children (80 males and 69 females). We measured children’s MA, word reading (WR), and RC from T1 to T4, in addition to phonological awareness, vocabulary knowledge, and general cognitive ability at T1 as control measures. Four plausible cross-lagged models were assessed and compared to examine the direction of the developmental relationships between MA and RC over time. Results found support for a reciprocal-causation model, that is, MA stably predicted subsequent RC, and the reverse relation was also found. Longitudinal mediation analyses revealed that WR partially mediated the relationship between MA and RC in Chinese children. These findings extend our understanding of the relationship between MA and RC. The practical implications for these two developing skills in Chinese children are discussed.

## Introduction

Morphological awareness (MA) is defined as the ability to reflect upon and manipulate morphemes (Kuo and Anderson, 2006). MA makes a unique contribution to reading comprehension (RC) in English, even after controlling for general intellectual ability, phonological awareness (PA), vocabulary, and word reading (WR; e.g., Nagy et al., 2006; Kirby et al., 2012). As the unique morphology of Chinese (Packard, 2000), MA is salient for Chinese children’s language and literacy development (McBride-Chang et al., 2003; Liu et al., 2013; Zhang et al., 2014; Cheng et al., 2015). Studies on Chinese reading development suggest that MA is important for RC beyond PA, vocabulary, and WR (Shu et al., 2006; Tong et al., 2009; Ho et al., 2012; Zhang et al., 2012; Yeung et al., 2013). Moreover, there is growing evidence that the relationship between MA and RC would be reciprocal (Kruk and Bergman, 2013; Deacon et al., 2014). However, the finding was from Indoeuropean languages, with limited research on the reciprocal relationship between these two skills in Chinese (for exception, Wu et al., 2009). Consequently, the present study was conducted with the aim of examining the longitudinal relationship between MA and RC among Chinese children.

Although, there is evidence of a correlation between MA and RC, the underlying mechanisms are unclear. MA is moderately correlated with WR. According to the Simple View of Reading (Hoover and Gough, 1990), RC is a product of decoding and linguistic comprehension (R = D × LC). Joshi et al. (2012) explored the SVR model among 102 Chinese children from grades 2 and 106 children from grade 4. Results indicated that WR, as measured by character recognition accounted for 22 and 32% of the variance in RC at grades 2 and 4 respectively. Thus, in the present study, we examined whether WR mediates the association between MA and RC in Chinese young children.

Before reviewing the relationship between MA and RC, the main characteristics of Chinese morphology were first briefly introduced. The orthography of Chinese is morphosyllabic, meaning that morphemes are in one-to-one correspondence with the basic writing unit – the character, or syllable. Compared to alphabetic languages, both inflectional and derivational morphology are rare in Chinese. There are three main characteristics of Chinese morphology. First, more than 70% of Chinese words are compounds, comprising two or three morphemes (Packard, 2000), whereas inflectional and derivational words are more productive than compounds in English. Compounding awareness is particularly important for Chinese reading development (Liu and McBride-Chang, 2010). Given the contrast of morphology between Chinese and other alphabetic languages (e.g., English), research on compound morphology in Chinese is of interest for understanding reading development across languages. Second, there are a large number of homophones in Chinese (Shu, 2003). Homophone awareness helps children distinguish the morphemes of homophonic characters in learning to read (McBride-Chang et al., 2003). Third, there are a large number of homonyms in Chinese (Li et al., 2002). A homonymic character may represent more than one morphemes. For instance, the homonymic character 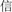 (xin 4) could correspond to several different meaning, including *letter, information, faithfulness, credit, at random*, and *a Chinese surname*. Homonym awareness is important for distinguishing homonymic characters meaning (Li et al., 2002; Liu et al., 2013).

Following with previous studies (e.g., Li et al., 2002; McBride-Chang et al., 2003; Liu et al., 2013), the comprehensive model of Chinese MA conceptualizes the three indicators as described above: compounding awareness, homophone awareness, and homonym awareness. Therefore, in the present study, we took this model into account. In other words, a latent MA was extracted using these three measures as indicators.

The present study used a longitudinal data set to examine the relationship between MA and RC among Chinese children. MA is a significant predictor of RC given the significant characteristics of Chinese orthography (McBride, 2016). There are several reasons why MA could directly affect RC in Chinese (Kuo and Anderson, 2006; McBride, 2016). First, Chinese is highly productive in compounding, and its compounding rules are relatively transparent and straightforward. There are no orthographic shifts in Chinese, and there are few pronunciation shifts when complex words are formed. Thus, once the meaning of a given morpheme is understood, the meaning of other novel compound words that contain the morpheme can be easily grasped. This can be helpful for activating background knowledge about the morpheme in a RC situation (Zhang et al., 2012). Second, given the one-to-one correspondence among syllables, morphemes, and characters in Chinese, children with heightened sensitivity to morphemes in the oral language have a better chance of success in learning to read (Chen et al., 2009). Due to the great number of homophones and homonyms in Chinese, it is important to understanding that one syllable may represent various meanings (Shu et al., 2006; Liu et al., 2013). The Chinese child who is making good use of morphological information will enable them to understand how morphemes fit together in Chinese speech and print, and how to differentiate across meanings in a RC situation, thus facilitating understanding of the text (Liu et al., 2013). It is important for higher level meaning-making process such as text comprehension (Pan et al., 2015).

The contribution of MA to Chinese children’s RC was established in several previous studies (Shu et al., 2006; Tong et al., 2009; Ho et al., 2012; Zhang et al., 2012; Yeung et al., 2013). In a 1-year longitudinal study with Cantonese-speaking children in kindergarten to grade 1, regression analysis results demonstrated that MA, measured with compounding awareness and homophone awareness, was associated with concurrent and longitudinal RC at the sentence level with reading-related variables statistically controlled (Tong et al., 2009). In a two-wave longitudinal study with Hong Kong Chinese primary school students, Zhang et al. (2012) found that after controlling for WR, PA and speeded naming at Time 1, MA, measured with compounding awareness and homophone awareness, was uniquely associated with concurrent and subsequent RC.

From a theoretical view, with increased exposure to complex print experiences, it is possible that learning to read triggers consciousness of certain morphological features. Given children have more opportunities to encounter with printed words in text, there is a reason to speculate that reading experience could help them to identify the critical morphemes, improve the quality of lexical representations, and manipulate the morphological structure of spoken words (Kuo and Anderson, 2006). Children might learn about the morphological processing through their reading printed materials. The relationship between MA and RC could be reciprocal. To our knowledge, three recent studies directly focused on the temporal relationship between MA and RC (Wu et al., 2009; Kruk and Bergman, 2013; Deacon et al., 2014). Kruk and Bergman (2013) assessed English-speaking children from grades 1 to 3 semi-annually. Using multilevel modeling, results found the reciprocal relations which were indicated by MA at grade 1 predicting growth in RC between grades 1 and 3, and vice versa. Deacon et al. (2014) assessed English-speaking children longitudinally from grades 3 to 4. Using cross-lagged panel modeling, results found that children’s MA at grade 3 explained their gains in RC at grade 4, and their early RC predicted MA at grade 4. The results of these two studies found the reciprocal relations between MA and RC among English-speaking children. Wu et al.’s (2009) report on an intervention in morphology of characters and words for Chinese children suggested that MA has a unidirectional influence on literacy in early second grade. In addition, the results suggested a reciprocal relation in early third grade between MA and children’s literacy development. It is possible that the relationship emerge for older children, such as those studied by Deacon et al. (2014), but that such relationship is more difficult to detect for young children (as in Wu et al., 2009). The inconsistencies among the results of these three studies emphasize the need for further research and motivate the current study. Therefore, the main goal of the present study was to examine longitudinal relations between Chinese children’s MA and RC during the early elementary school years.

Word reading has also been shown to mediate the relationship between MA and RC in English-speaking children (Jarmulowicz et al., 2008; Deacon et al., 2014). Jarmulowicz et al. (2008) found that nonsense WR completely mediated the relationship between derivational MA and RC in third grade English-speaking children. In a longitudinal study, Deacon et al. (2014) revealed that WR skills partially mediated the relationship between MA and RC in grades 3 and 4. MA is moderately correlated with Chinese WR (Li et al., 2012; Liu et al., 2013) and is longitudinally associated with learning to read Chinese (e.g., Yeung et al., 2013). Li et al. (2012) administered a battery of reading-related tests (including several MA tasks such as compound and homophone interpretation) to kindergarteners and primary school students in mainland China. The results showed that MA accounted for substantial unique variance in character reading in first and fourth graders, after controlling for IQ and PA. A recent study (Liu et al., 2013) examined the associations of homophone awareness and compounding awareness to Chinese WR and vocabulary knowledge (VK) among Hong Kong Chinese children. The results showed that homophone awareness and compounding awareness were uniquely associated with WR, with autoregressors statistically controlled. In a 3-year longitudinal study, Yeung et al. (2013) found that MA assessed with a morphological construction task in grade 1 was a significant longitudinal predictor of Chinese WR in grades 1–4 after controlling for autoregressive effects. Given the emerging evidence of the importance of morphology in WR, a possibility is that MA facilitates efficient WR, which in turn boosts RC. Yeung et al. (2013) found that MA, measured by morpheme identification or homophone discrimination, had significant indirect effects on RC via WR in fourth graders in Hong Kong. Therefore, the second goal of the present study was to examine whether WR would mediate the relationship between Chinese children’s MA and RC.

**FIGURE 1:**
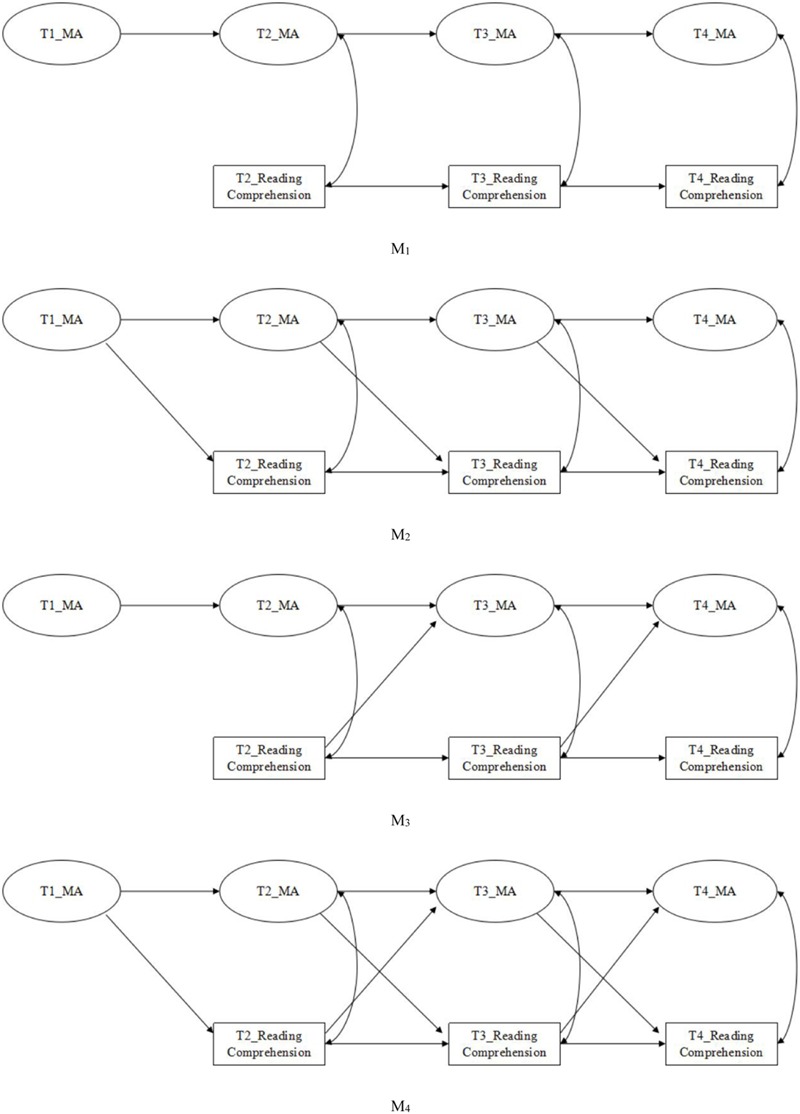
**All the tested models (M_1_–M_4_).** M_1_: a baseline model with stability (across-time) and synchronous (within-time) paths, and no cross-lagged paths; M_2_: a standard causal model with cross-lagged paths from prior morphological awareness (MA) to later reading comprehension (RC); M_3_: a reverse-causation model with cross-lagged paths from prior RC to later MA; M_4_: a reciprocal-causation model with both cross-lagged paths representing reciprocal effects. The measurement model and the control variables are not shown here to simplify the representation of the model. MA, morphological awareness.

A longitudinal cross-lagged panel model was recommended to comprehensively assess the relationship between MA and RC. The following four competing models were tested and compared (**Figure 1**): (a) a baseline model only with across-time stability paths (M_1_), (b) a standard causal model with cross-lagged paths from prior MA to later RC (M_2_), (c) a reverse-causation model with cross-lagged paths from prior RC to later MA (M_3_), and (d) a reciprocal-causation model with both cross-lagged paths (M_4_). Note that, paths were only estimated between adjacent time points after considering statistical power and the total number of parameters (Selig and Little, 2012). We concluded by assessing a final model of best fit between MA and RC. The second goal of the study was to investigate whether WR would mediate the relationship between MA and RC. A cross-lagged panel mediation model was conducted for longitudinal mediation analyses (Selig and Little, 2012). Several control variables, such as general cognitive ability, PA, and VK were included in the model. These control variables were selected due to reducing the possible effects of skills to be associated with children’s MA and RC (Ho et al., 2004; Kuo and Anderson, 2006; Joshi et al., 2012; Li et al., 2012).

## Materials and Methods

### Participants

This study is part of an ongoing longitudinal study (e.g., Cheng et al., 2016) which was approved by the relevant Research Ethics committee of Beijing Normal University. Written informed consent was obtained from school principals, classroom teachers and parents of all of these children for their child’s participation prior to the study.

One hundred and forty-nine first graders (80 boys; mean age at the first testing was 6.25 years and age range was 5.58—6.92 years) from two urban elementary schools in Shanxi, China were recruited and tested four times between grades 1 to 2: fall (Time 1; *n* = 149) and spring (Time 2; *n* = 146) of the first grade, and fall (Time 3; *n* = 127) and spring (Time 4; *n* = 128) of the second grade.

### Measures and Procedure

A battery of tests was administered by trained testers. These are described as follows and displayed by the time of administration in **Table 1**. Tests administered in groups were RC and general cognitive ability. Tests that were administered individually were compounding awareness, homophone awareness, homonym awareness, WR, PA, and VK.

**Table 1:** Means, standard deviations, skewness, kurtosis, and reliabilities of all measures from T1 to T4.

Measures	Time	*M* (*SD*)	Skewness	Kurtosis	Reliability (alpha)
Compounding awareness (60)	T1	9.57 (8.97)	1.22	1.38	0.83
	T2	15.36 (11.64)	0.76	-0.18	0.85
	T3	20.94 (10.81)	0.01	-0.70	0.80
	T4	25.87 (10.52)	-0.06	-0.45	0.78
Homophone awareness (22∖25∖29∖36)^a^	T1	6.79 (3.81)	1.04	2.83	0.90
	T2	8.68 (4.60)	0.65	0.11	0.87
	T3	13.39 (4.61)	0.79	1.09	0.72
	T4	13.23 (6.39)	0.94	1.04	0.84
Homonym awareness (24)	T1	5.88 (3.03)	0.36	0.08	0.70
	T2	9.26 (3.16)	0.11	-0.51	0.66
	T3	10.67 (2.94)	0.23	-0.15	0.78
	T4	12.09 (3.89)	-0.07	-0.36	0.71
Word reading (150)	T2	44.72 (24.93)	0.46	-0.55	0.99
	T3	59.65(23.50)	-0.02	-0.70	0.99
	T4	77.29 (20.73)	-0.31	-0.32	0.99
Reading comprehension (18)	T2	7.73 (3.26)	-0.03	-0.51	0.66
	T3	9.06 (3.99)	0.24	-0.60	0.79
	T4	11.06 (4.02)	-0.31	-0.75	0.81
Phonological awareness (12)	T1	6.05 (3.83)	-0.06	-1.22	0.88
Vocabulary knowledge (64)	T1	8.62 (5.14)	0.38	-0.54	0.74
General cognitive ability (60)	T1	28.06 (9.31)	-0.16	-0.91	0.91

*Ns = 127 – 149. Numbers in parentheses represent the maximum score for each measure. ^a^Numbers in parentheses were the maximum score that was given for four measures. They were not necessarily the maximum score due to the open-ended nature of the morphological homophone awareness task. T1 = fall of first grade; T2 = spring of first grade; T3 = fall of second grade; T4 = spring of second grade.*

### Morphological Awareness (MA)

Morphological awareness was assessed with three tasks at each time point from T1 to T4. The three MA tasks did not involve reading or writing at all.

#### Compounding Awareness (CA)

Compound production task, which has been used in previous studies (e.g., Cheng et al., 2015), requires children to produce an appropriate word based on an orally presented question/scenario (e.g., “What should we call a bird like a frog?” The best answer would be *“*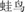 *wa1 niao3, frog-bird”*). Children’s responses were rated by two trained psychology majors on a scale of 0–3 based on the rating criteria (Cheng et al., 2015). Three points were allotted for the response that included all critical morphemes and a correct and succinct structure (e.g., 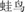, *wa1 niao3, frog bird, for the above example)*. Two points were for a response that included all critical morphemes and a correct but partially redundant structure (e.g., 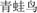 *qing1 wa1 niao3, green frog bird*, where 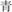 *qing1 green* is redundant, or 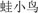 *wa1 xiao3 niao3, frog little bird*, where 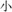 *xiao3 little* is redundant, for the above example). One point was for a response that included all critical morphemes and a correct but completely redundant structure (e.g., 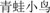 *qing 1 wa1 xiao3 niao3, green frog little bird* where 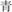 *qing1* and 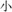 *xiao3* are redundant, for the previous example). Zero point was for a response with missing critical morphemes, a totally unrelated response or no response (e.g., 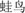 *qing 1 niao3, green bird* where the critical morpheme 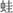 *wa1*, is missing, for the previous example). Testing stopped when children gave 0-point responses for five consecutive items. The inter-rater reliability for each time point was 0.95, 0.97, 0.96, and 0.96, respectively. This task contained eight practice items and 20 test items.

#### Homophone Awareness (HPA)

The morphological homophone awareness task (Liu and McBride-Chang, 2010) consisting of 12 items was administered to assess children’s homophone awareness. The target morpheme was orally presented together with a sample word containing it. After that, children were asked to name another word that contained the target morpheme and then asked to name words containing the homophones of the target morpheme as many as possible. For example, the target morpheme/yue4/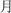 (moon) in /yue4liang4/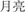 (moon) was orally presented to the children. Children were then asked to produce words with the same /yue4/ pronunciation, such as /yue4/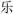 in /yin1yue4/ 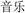 (music), /yue4/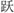 in /tiao4yue4/ 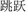 (jump). Child was encouraged to name as many words as possible. 1 mark was given for each different homophone. The scores were obtained based on the number of different homophone morphemes produced. Thus, there was no maximum score.

#### Homonym Awareness (HNA)

Children’s homonym awareness was assessed with morpheme production task which has been used in previous studies (e.g., Shu et al., 2006). In this task, children were orally presented a two-syllable Chinese word in which one morpheme was identified, and then were required to produce two words with the target morpheme. The morpheme in one word should have the same meaning as the target morpheme while the other should have a different meaning from the target morpheme. However, both morphemes were identical in pronunciation. For example, children were given the target morpheme /ming2/ 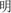 in /ming2tian1/ 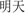 (tomorrow), and then were required to create two words with the morpheme /ming2/. The morpheme /ming2/ in one word was supposed to have the same meaning as it did in /ming2tian1/. One possible answer would be /ming2nian2/ 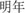 (next year). The morpheme /ming2/ in another word was supposed to have the different meaning from that in /ming2tian1/. One acceptable answer would be /ming2liang4/ 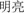 (brightness). A simple example in English would be that the *light* in *stoplight* has the same meaning as the one in *lightbulb*, and a different meaning from the one in *light-headed*. All items consisted of real words. This task consisted of two practice items and 12 test items.

### Reading Comprehension (RC)

The RC task (Mo, 2004), which has been used in previous studies (Joshi et al., 2012), requires children to read one narrative passage and answer 18 multiple-choice questions. It was administered at T2, T3, and T4. We did not measure RC at T1 given the children’s young ages and reading experiences. This task is age appropriate for first-grade and second-grade Chinese students and has good reliability and validity. The task was not timed. The title of this passage was *Prince Nezha Conquers the Dragon King* (selected from Journey to the West by Wu Cheng’en). There were three kinds of questions: (1) focusing on the particular words and phrases contained in the text (seven questions), (2) focusing on the global topics and the interrelationship (nine questions), and (3) focusing on integration of ideas and information provided by the text with relevant prior knowledge (two questions). One sample question was “*According to the sentence, ‘Nezha made up his mind to punish them and fight for ordinary people,’ which one of the following can be inferred?” (A) He was very naughty. (B) He sympathized with ordinary people. (C) He wanted to try his two weapons. (D) He fought for himself.*” One point was awarded for each correct answer.

### Word Reading (WR)

Word reading was assessed with character recognition task at each time point from T2 to T4. Chinese character recognition task, which has been used in previous studies (e.g., Li et al., 2012), requires children to read the characters from the beginning and stop when they failed to read 15 consecutive items. The task consisted of 150 single characters with increasing difficulty.

### Control Variables

Control variables including PA, VK, and general cognitive ability were assessed at T1 only.

#### Phonological Awareness (PA)

Phoneme deletion task (Shu et al., 2006) requires children to produce a new syllable by deleting one phoneme from a monosyllabic Chinese word (e.g., /cha1/, with the /ch/ taken away would be /a1/). It consisted of four practice items and 12 test items.

#### Vocabulary Knowledge (VK)

Vocabulary definitions task, which was adapted for Mandarin Chinese-speaking children from the Stanford–Binet Intelligence Scale vocabulary subtest and has been used in previous studies (e.g., Song et al., 2015), requires children to orally explain or define 32 words which were arranged in order of increasing difficulty.

#### General Cognitive Ability

Children’s general cognitive ability (non-verbal IQ) was assessed with Raven’s Standard Progressive Matrices (Zhang and Wang, 1985). The test contained 60 items. The maximum score on the Raven’s Matrices was 60.

## Results

### Attrition Analysis

The attrition rate from T1 to T4 was 14%. We conducted a series of *t*-test to investigate whether the children who remained in the study (*n* = 128) differed from those who dropped out of the study (*n* = 21) on all measures at T1. The results showed no significant differences on any measure (*p*s > 0.05). Little’s missing completely at random test (Little, 1988) showed data was missing completely at random (MAR), χ^2^(*df* = 92) = 79.89, *p* = 0.81. Missing data were handled using full-information maximum likelihood under the MAR assumption in Mplus 7.11 (Muthén et al., 1998–2012) during model estimation.

### Preliminary Analyses

**Table 1** presented means, standard deviations, skewness, kurtosis, and the internal consistency of all measures over time. Obvious increases with time were observed in compounding awareness, homophone awareness, and homonym awareness.

**Table 2** presented correlations among all measures over time. The three tasks of MA showed moderate temporal stability across time. Correlations between the three tasks of MA at each time point and RC at T3 and T4 were also significant except for homophone awareness at T1 and RC at T3.

**Table 2:** Correlations among all variables.

	1	2	3	4	5	6	7	8	9	10	11	12	13	14	15	16	17	18	19	20
(1) T1 CA	1																			
(2) T2 CA	0.64^∗∗^	1																		
(3) T3 CA	0.60^∗∗^	0.65^∗∗^	1																	
(4) T4 CA	0.47^∗∗^	0.62^∗∗^	0.70^∗∗^	1																
(5) T1 HPA	0.32^∗∗^	0.26^∗∗^	0.29^∗∗^	0.25^∗∗^	1															
(6) T2 HPA	0.26^∗∗^	0.41^∗∗^	0.35^∗∗^	0.44^∗∗^	0.30^∗∗^	1														
(7) T3 HPA	0.35^∗∗^	0.26^∗∗^	0.37^∗∗^	0.40^∗∗^	0.26^∗∗^	0.56^∗∗^	1													
(8) T4 HPA	0.37^∗∗^	0.40^∗∗^	0.48^∗∗^	0.53^∗∗^	0.27^∗∗^	0.58^∗∗^	0.63^∗∗^	1												
(9) T1 HNA	0.44^∗∗^	0.44^∗∗^	0.39^∗∗^	0.34^∗∗^	0.26^∗∗^	0.27^∗∗^	0.35^∗∗^	0.33^∗∗^	1											
(10) T2 HNA	0.29^∗∗^	0.38^∗∗^	0.35^∗∗^	0.39^∗∗^	0.23^∗∗^	0.42^∗∗^	0.28^∗∗^	0.36^∗∗^	0.35^∗∗^	1										
(11) T3 HNA	0.38^∗∗^	0.38^∗∗^	0.50^∗∗^	0.40^∗∗^	0.37^∗∗^	0.44^∗∗^	0.46^∗∗^	0.47^∗∗^	0.35^∗∗^	0.45^∗∗^	1									
(12) T4 HNA	0.39^∗∗^	0.37^∗∗^	0.45^∗∗^	0.56^∗∗^	0.23^∗∗^	0.38^∗∗^	0.42^∗∗^	0.55^∗∗^	0.31^∗∗^	0.46^∗∗^	0.45^∗∗^	1								
(13) T2 WR	0.34^∗∗^	0.44^∗∗^	0.39^∗∗^	0.42^∗∗^	0.21^∗^	0.34^∗∗^	0.42^∗∗^	0.42^∗∗^	0.33^∗∗^	0.30^∗∗^	0.39^∗∗^	0.33^∗∗^	1							
(14) T3 WR	0.29^∗∗^	0.38^∗∗^	0.43^∗∗^	0.42^∗∗^	0.24^∗∗^	0.31^∗∗^	0.41^∗∗^	0.48^∗∗^	0.28^∗∗^	0.30^∗∗^	0.37^∗∗^	0.39^∗∗^	0.89^∗∗^	1						
(15) T4 WR	0.34^∗∗^	0.43^∗∗^	0.49^∗∗^	0.48^∗∗^	0.29^∗∗^	0.34^∗∗^	0.34^∗∗^	0.48^∗∗^	0.24^∗∗^	0.27^∗∗^	0.37^∗∗^	0.38^∗∗^	0.80^∗∗^	0.92^∗∗^	1					
(16) T1 PA	0.14	0.09	0.26^∗∗^	0.30^∗∗^	0.12	0.27^∗∗^	0.29^∗∗^	0.37^∗∗^	0.27^∗∗^	0.17^∗^	0.25^∗∗^	0.17	0.18^∗^	0.21^∗^	0.21^∗^	1				
(17) T1 VK	0.42^∗∗^	0.47^∗∗^	0.47^∗∗^	0.46^∗∗^	0.25^∗∗^	0.41^∗∗^	0.45^∗∗^	0.47^∗∗^	0.47^∗∗^	0.53^∗∗^	0.45^∗∗^	0.39^∗∗^	0.27^∗∗^	0.31^∗∗^	0.34^∗∗^	0.29^∗∗^	1			
(18) T1 IQ	0.28^∗∗^	0.30^∗∗^	0.28^∗∗^	0.28^∗∗^	0.07	0.27^∗∗^	0.21^∗^	0.21^∗^	0.26^∗∗^	0.36^∗∗^	0.23^∗∗^	0.25^∗∗^	0.30^∗∗^	0.24^∗∗^	0.27^∗∗^	0.18^∗^	0.40^∗∗^	1		
(19) T2 RC	0.09	0.10	0.12	0.14	-0.03	-0.01	0.15	0.12	0.10	-0.02	0.16	0.05	0.40^∗∗^	0.42^∗∗^	0.36^∗∗^	0.15	0.15	0.24^∗∗^	1	
(20) T3 RC	0.38^∗∗^	0.44^∗∗^	0.51^∗∗^	0.51^∗∗^	0.08	0.31^∗∗^	0.27^∗∗^	0.44^∗∗^	0.33^∗∗^	0.31^∗∗^	0.35^∗∗^	0.30^∗∗^	0.63^∗∗^	0.63^∗∗^	0.59^∗∗^	0.32^∗∗^	0.39^∗∗^	0.31^∗∗^	0.44^∗∗^	1
(21) T4 RC	0.33^∗∗^	0.39^∗∗^	0.47^∗∗^	0.51^∗∗^	0.20^∗^	0.31^∗∗^	0.27^∗∗^	0.45^∗∗^	0.32^∗∗^	0.36^∗∗^	0.32^∗∗^	0.36^∗∗^	0.47^∗∗^	0.56^∗∗^	0.59^∗∗^	0.26^∗∗^	0.37^∗∗^	0.39^∗∗^	0.29^∗∗^	0.64^∗∗^

*Ns = 127 – 149. CA, compounding awareness; HPA, homophone awareness; HNA, homonym awareness; WR, word reading; PA, phonological awareness; VK, vocabulary knowledge; RC, reading comprehension. ^∗^*p* < 0.05, ^∗∗^*p* < 0.01, two-tailed.*

To ensure unbiased effects of the measures across time points, longitudinal measurement invariance was examined following recommended procedures by Cheung and Rensvold (2002) and Brown (2006). To evaluate the model fit, we reported chi-square values (χ^2^), comparative fit index (CFI), Tucker-Lewis index (TLI), root mean square error of approximation (RMSEA), and standardized root mean square residual (SRMR). According to recommendations by several researchers (Hu and Bentler, 1999; Kline, 2011), in broad strokes, CFI and TLI ≥ 0.90, RMSEA ≤ 0.06, and SRMR ≤ 0.08 are considered as good fit. Model comparison for invariance analysis relies on the chi-square difference test (Δχ^2^) (Cheung and Rensvold, 2002; Kline, 2011). Following generally accepted practice, the hypothesis of invariance over time is rejected when the chi-square difference test (Δχ^2^) between nested models has a probability lower than 0.05 (*p* of Δχ^2^ ≤ 0.05).

The baseline model of non-invariance was first specified in which the loadings were allowed to vary completely. This model showed good fit, χ^2^(*df* = 30) = 26.58, *p* = 0.65 (χ^2^/*df* = 0.89), CFI = 1.00, TLI = 1.00, RMSEA = 0.00 (90% CI = 0.00–0.05), and SRMR = 0.03. When a full invariance model was fit, it was a non-significant loss of fit compared to the non-invariance model (Δχ^2^ = 10.27, Δ*df* = 6, *p* = 0.11), χ^2^(*df* = 36) = 36.85, *p* = 0.30 (χ^2^/*df* = 1.02), CFI = 0.99, TLI = 0.99, RMSEA = 0.03 (90% CI = 0.00–0.07), and SRMR = 0.06.

### The Relationship between Morphological Awareness and Reading Comprehension

Cross-lagged panel model was used to test the fit of four competing models (M_1_ to M_4_, see **Figure 1**) of the developmental relationship between MA and RC over a total of four time points, while controlling for the other cognitive and linguistic variables. Chi square difference testing was also conducted to compare the model fits.

In the theoretical model, the relations between variables are assumed to be stable across time by constraining the autoregressive and cross-lagged paths to be equal. In **Figure 1**, autoregressive effects in M_1_–M_4_ were constrained to be equal. That is, the paths for MA between T1 and T2, T2 and T3, T3 and T4 were constrained to be equal during model estimation. And also, the paths for RC between T2 and T3, T3 and T4 were constrained to be equal during model estimation. Besides, the cross-lagged effects, which were paths of MA for prospectively predicting RC (in M_2_ and M_4_) and of RC for prospectively predicting MA (in M_3_ and M_4_), were constrained to be equal respectively during model estimation. Additionally, we also calculated synchronous (within-time) covariances between MA and RC in the model. PA, VK, and general cognitive ability (IQ) were included as control variables during model estimation. All control variables were permitted to correlate with each other, and all control variable paths to MA and RC at T2, T3, and T4 were calculated.

**FIGURE 2:**
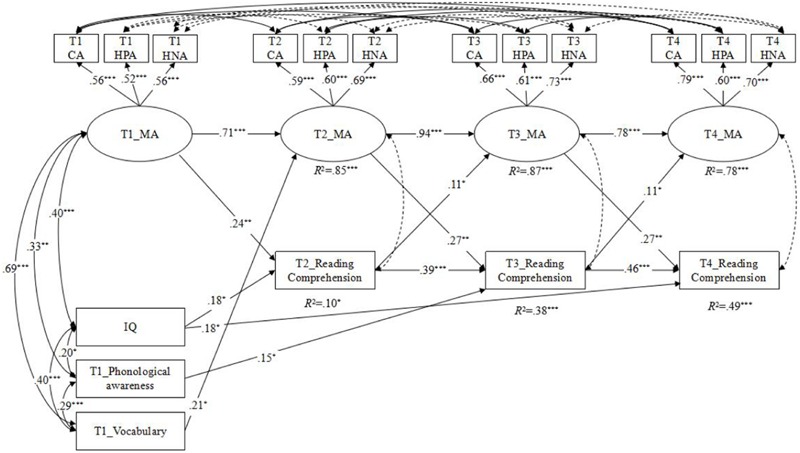
**The final model (*M_4_*) with a completely standardized solution to address the relationship between MA and RC after controlling for phonological awareness, vocabulary knowledge, and general cognitive ability.** MA, morphological awareness; CA, compounding awareness; HPA, homophone awareness; HNA, homonym awareness. Dashed lines indicate non-significant paths. ^∗^*p* < 0.05, ^∗∗^*p* < 0.01, ^∗∗∗^*p* < 0.001, two-tailed.

As shown in **Table 3**, the standard causal model (M_2_) (Δχ^2^ = 9.80, Δ*df* = 1, *p* < 0.05), the reverse-causation (M_3_) (Δχ^2^ = 5.21, Δ*df* = 1, *p* < 0.05) and the reciprocal-causation model (M_4_) (Δχ^2^ = 14.28, Δ*df* = 2, *p* < 0.05), were significantly different from the baseline stability model (M_1_). Besides, there was significant difference between the standard causal model (M_2_) and the reciprocal-causation model (M_4_) (Δχ^2^ = 4.48, Δ*df* = 1, *p* < 0.05), indicating that the fit of the reciprocal-causation model (M_4_) was more satisfactory. Consequently, the reciprocal-causation model (M_4_) was accepted as the best model. **Figure 2** reports the standardized path coefficients for this model.

**Table 3:** Fit indices and model comparisons among competing models to address the relationship between morphological awareness and reading comprehension.

	Fit indices					
Tested models	χ^2^	Df	RMSEA (90%CI)	SRMR	CFI	TLI
Baseline model (M_1_)	139.39	100	0.051 [0.029,0.071]	0.078	0.957	0.936
Standard causal model (M_2_)	129.59	99	0.046 [0.019,0.066]	0.065	0.967	0.949
Reverse-causation model (M_3_)	134.18	99	0.049 [0.025,0.069]	0.077	0.962	0.942
Reciprocal-causation model (M_4_)	125.11	98	0.043 [0.013,0.064]	0.064	0.970	0.955

Model comparisons	*Δ*χ^2^	*Δ df*	*p*			

M_2_ vs. M_1_	9.80	1	0.01			
M_3_ vs. M_1_	5.21	1	0.02			
M_4_ vs. M_1_	14.28	2	0.01			
M_4_ vs. M_2_	4.48	1	0.03			
M_4_ vs. M_3_	9.07	1	0.01			

*RMSEA, root-mean-square error of approximation; CI, confidence interval; SRMR, standardized root-mean-square residual; CFI, comparative fit index; TLI, Tucker-Lewis fit index.*

As can be seen in **Figure 2**, the stability paths for MA and RC were significant. The cross-lagged paths from MA at T1 to RC at T2 (standardized β = 0.24, *p* < 0.01), from MA at T2 to RC at T3 (standardized β = 0.27, *p* < 0.01), from MA at T3 to RC at T4 (standardized β = 0.27, *p* < 0.01), were all significant even after controlling for the other cognitive and linguistic covariates, indicating that the effect of MA on RC was stable across time. And also the cross-lagged paths from RC at T2 to MA at T3 (standardized β = 0.11, *p* < 0.05) and from RC at T3 to MA at T4 (standardized β = 0.11, *p* < 0.05) were both significant even after controlling for the other cognitive and linguistic covariates, indicating that the effect of RC on MA was stable across time.

### Longitudinal Mediation of Word Reading between Morphological Awareness and Reading Comprehension

**FIGURE 3:**
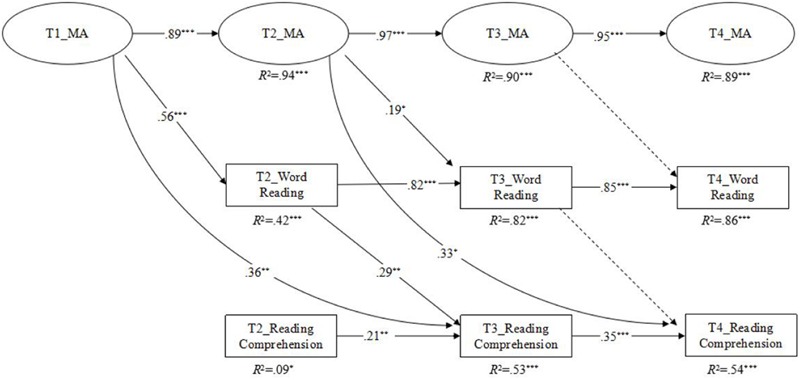
**The longitudinal mediation model to address the mediation of word reading in the relationship between MA and RC.** The measurement model and the control variables are not shown here to simplify the representation of the model. MA, morphological awareness. Dashed lines indicate non-significant paths. ^∗^*p* < 0.05, ^∗∗^*p* < 0.01, ^∗∗∗^*p* < 0.001, two-tailed.

Longitudinal mediation model was conducted to investigate whether WR mediates the relationship between MA and RC. As can be seen in **Figure 3**, results found that MA at T1 made significant indirect contributions to RC at T3 via WR at T2 in addition to a significant direct contribution. To evaluate the indirect contribution of MA on RC via WR, bias-corrected bootstrapped standard errors were calculated for the parameters and used to construct 95% confidence intervals to determine whether the mediated effects were statistically significant. Results indicated that MA made significant indirect contributions to RC via WR (standardized β = 0.16, 95% CI [0.06, 0.48]).

## Discussion

Cross-lagged panel model was conducted to examine the temporal sequence of MA and RC among Chinese-speaking children. The present results found that children’s MA stably predicted later RC across grades 1–2. In the other direction, children’s RC was a predictor of later MA. The findings are consistent with conclusions offered by previous studies to the effect that MA uniquely contributes to RC among Chinese children (Shu et al., 2006; Tong et al., 2009; Ho et al., 2012; Zhang et al., 2012; Yeung et al., 2013). Moreover, our results replicate and extend findings from the two-wave longitudinal study (Tong et al., 2009; Zhang et al., 2012). To our knowledge, longitudinal studies of MA were limited. Cross-lagged panel analysis for longitudinal data has allowed us to identify the stable contribution of MA to RC. Our findings suggest that there is a positive longitudinal effect of MA on RC over and above continuity. Results of the present study extend the important role of MA for RC among Chinese children by demonstrating its importance stably.

Theoretically, it would seem that exposure to print is related to MA. Thus, we had expected the reciprocal relationship between MA and RC. The present study demonstrated that RC was a predictor of MA across grades 1–2. Our results are consistent with the prior studies that have addressed the temporal relationship between MA and RC (Wu et al., 2009; Kruk and Bergman, 2013; Deacon et al., 2014). More specifically, the current study goes beyond the study by Wu et al. (2009) by extending the reciprocal relationship to the children across grades 1–2. It seems that children who have more reading experiences can use their understanding of texts better to gain more from their reading experiences (Wu et al., 2009; Deacon et al., 2014). In other words, the reading experiences provides an opportunity for children to develop abstract understanding of key morphemes (homophones and homonyms) and extract the structure of compounding word, and then support the growth of MA. As children’s literacy and reading development, the relationships between MA and RC become cumulatively reciprocal. In other words, children develop the skills needed to form a coherent mental representations of a text, including understanding the literal meaning of specific words and syntax structures, inferring the meaning of novel words, building a mental representation of people, states, settings, actions, and events that are either explicitly mentioned or inferred from text. Children who have enough reading experience might be able to learn and extract the meaning of morphologically complex words which they encounter in text. Children might better identify and understand the critical morpheme in reading process. It seems that MA and RC are on the basis of each other and developmentally intertwined.

The second goal of the study was to examine whether WR would mediate the relationship between MA and RC. The findings of the present study revealed significant indirect effects of MA on RC via WR. Chinese is relatively semantically transparent at both the lexical and sub-character levels. MA involves the integration of semantic, phonological, and syntactic information, which provides insights into semantic information during real-time RC (Perfetti et al., 2005; Kuo and Anderson, 2006). First, as the form of lexical compounding awareness, children’s ability of morphological analysis makes them decompose complex words and characters to infer the meaning of morphologically complex words. They can use the extra cues to learn and extract the meaning of morphologically complex words, thereby facilitating understanding the gist of the meaning of a text (Kuo and Anderson, 2006; Liu et al., 2013). In addition, children’s MA allows them to infer the meaning of critical morphemes to avoid confusing many homophones and homonyms better, and this appears to be helpful for comprehending text. Children can distinguish and discriminate meanings to ensure understandings in reading by means of homophone awareness (Zhang et al., 2014). Children who make good use of homophone awareness can produce more morphemes that sound the same. This enables them to identify the meanings of the homophones because phonological cues of Chinese are relatively less unreliable. Third, children who make good use of homonym awareness may disambiguate a character by focusing on the different semantic representations of the character in context, thus facilitating text comprehension. Fourth, given the absence of word boundaries, MA can serve as a cue in reading Chinese (Lin et al., 2011). MA might foster flexibility in making use of context for understanding sentences or texts. Children with more-developed MA are better at distinguishing the appropriate words meanings in reading process. In this sense, MA affects WR or understanding of word meaning, which in turn influences RC.

Given the difference in the process of reading in Chinese and English, research on morphology in Chinese is of interest for understanding the universality and specificity of the role of morphology in reading development across languages because most of the literature on morphology in English has focused on inflectional and deviational morphology.

Certain drawbacks of the present study suggest additional avenues for future research. First, we did not take into account children at middle or high elementary school years, the period known to be critical in the development of reading ability. Of course this will be a longer period under longitudinal set up. More studies are needed to examine the developmental relationship between MA and RC among children in later grades as more exposure to print. Second, the cross-lagged research design could not make causal inferences (Selig and Little, 2012). We should conduct experimental or intervention studies to test the temporal order of the relationship between MA and RC in future research. Third, we used only one passage in the RC task. The inclusion of multiple genres and passages across testing time points would enable the assessment of multiple constructs of RC. Fourth, there was the possibility of slight floor effects in several measures, particularly at Time 1, which would have limited variance in these variables to some extent. This may suppress the predictive effect of MA on RC and suggests that multiple measures of MA should be used in future studies.

The limitations of the current study notwithstanding, the present study has certain strengths. First, unraveling the direction of developmental relationships between MA and RC may contribute to theories of Chinese literacy acquisition. Second, our findings may shed light on the instructional practices for early children’s language and reading developments. Hence, we argue that to make MA instruction aimed at improving their RC performance. Because there is so much lexical compounding, compounding awareness appears to be particularly salient for Chinese children’s reading development (Liu and McBride-Chang, 2010; Liu et al., 2013; Cheng et al., 2016), future interventions could focus on developing children’s compounding awareness.

## Author Contributions

Conception and design of the study: YC, JZ, XW, HLi; acquisition, analysis, and interpretation of data: YC, JZ, XW, HLiu, HLi; drafting the work and revising it critically for important intellectual content: YC, JZ, XW, HLi.

## Conflict of Interest Statement

The authors declare that the research was conducted in the absence of any commercial or financial relationships that could be construed as a potential conflict of interest.

The reviewer HB and the handling Editor declared their shared affiliation, and the handling Editor states that the process nevertheless met the standards of a fair and objective review.
